# Effects of Fucoidan on the Inhibition of Biofilm Formation of *Salmonella enterica* Subsp. *enterica* Serovar *Typhimurium* on Seafoods and Its Molecular Antibiofilm Mechanisms

**DOI:** 10.3390/microorganisms13040914

**Published:** 2025-04-16

**Authors:** Anamika Roy, Pantu Kumar Roy, Sung Rae Cho, Shin Young Park

**Affiliations:** 1 Department of Seafood Science and Technology, Institute of Marine Industry, Gyeongsang National University, Tongyeong 53064, Republic of Korea; anamika32roy@gmail.com; 2 South Sea Fisheries Research Institute, National Institute of Fisheries Science, Yeosu 59780, Republic of Korea; srchoi1113@korea.kr

**Keywords:** biofilms, *Salmonella enterica* subsp. *enterica* Serovar *Typhimurium*, crab shells, shrimp shells, fucoidan

## Abstract

Foodborne illnesses, particularly those caused by *Salmonella enterica* subsp. *enterica* Serovar *Typhimurium*, present a significant challenge to public health, especially within the seafood industry due to biofilm formation on foods. This study investigated the antibiofilm potential of fucoidan, a sulfated polysaccharide, against *Salmonella enterica* subsp. *enterica* Serovar *Typhimurium* biofilm on crab and shrimp surfaces. Fucoidan’s minimum inhibitory concentration (MIC) was determined to be 150 µg/mL. Sub-MIC (1/8, 1/4, 1/2, and MIC) were evaluated for their impact on inhibition of biofilm formation. Fucoidan treatment resulted in significant, dose-dependent inhibition in biofilm formation, achieving 2.61 log CFU/cm^2^ and 2.45 log CFU/cm^2^ reductions on crab and shrimp surfaces, respectively. FE-SEM analysis confirmed biofilm disruption and cell membrane damage. Real-time PCR showed the downregulation of quorum-sensing (*luxS*) and virulence (*rpoS*, *avrA*, and *hilA*) genes. These results propose that fucoidan has the ability as a natural antibacterial agent for controlling *Salmonella enterica* subsp. *enterica* Serovar *Typhimurium* biofilms in seafood processing, thereby enhancing food safety and minimizing contamination.

## 1. Introduction

Foodborne infections are caused by eating tainted food, and their increasing prevalence is a serious public health issue [1,2]. Biofilm formation on seafood surfaces compromises food safety and raises the cross-contamination risk during processing, posing a direct threat to public health [3,4]. Bacterial cells permanently attached to food and food contact surfaces and immersed in a self-made matrix of extracellular polymeric substances (EPS) are recognized as biofilms [5]. *Salmonella* spp. is a pathogenic bacterium known to cause foodborne illness [6]. The Centers for Disease Control and Prevention (CDC) estimated that salmonellosis leads to infections of 1.35 million, 26,500 hospitalizations, and 420 fatalities each year in the United States [7]. The rise in salmonellosis cases has been attributed to *Salmonella* spp. biofilms on various food products and food-contact surfaces within food processing facilities [1]. Compared to planktonic cells, bacteria in biofilms can better adapt to various environmental situations. Biofilms are challenging to eliminate, representing a substantial hygienic concern in the seafood industry [8]. These biofilms on biotic and abiotic surfaces impact the safety and quality of food products. Effectively controlling biofilm formation remains a major challenge in the food sector, as they contribute to food contamination, damage processing surfaces and equipment, and pose serious health risks [3]. Biofilms are responsible for 80% of persistent bacterial infections, making it increasingly difficult to eliminate these pathogens [9]. As many chronic infections stem from biofilms, finding effective treatments has become a significant challenge [5,10]. Recently, intense research has been engaged on the expansion of new pharmaceuticals with improved efficacy, with a particular emphasis on innovative approaches and technological advancements. *Salmonella enterica* subsp. *enterica* Serovar *Typhimurium* is known for its ability to form robust biofilms, which promote its persistence in the environment and its resistance to conventional treatments [6,11]. In the seafood industry, biofilm formation by pathogenic bacteria, such as *Salmonella enterica* subsp. *enterica* Serovar *Typhimurium* on the surfaces of crabs and shrimps presents a significant challenge to food safety and public health.

Quorum sensing (QS) and extracellular polymeric substances (EPS) play critical roles in biofilm formation, stability, and pathogenicity in many bacteria, including *Salmonella Typhimurium* and *Listeria monocytogenes* [4]. QS is a cell-to-cell communication mechanism that regulates bacterial behavior in a population-density-dependent manner through signaling molecules known as autoinducers. In *S. Typhimurium*, the *luxS* gene is involved in the synthesis of autoinducer-2 (AI-2), a key signal in QS [6]. QS modulates the expression of numerous genes associated with virulence, motility, and biofilm development, facilitating bacterial adaptation and survival in diverse environments.

EPS, the major component of the biofilm matrix, is composed of polysaccharides, proteins, lipids, and extracellular DNA. It provides structural integrity to the biofilm, mediates adhesion to surfaces, offers protection against environmental stressors and antimicrobial agents, and hosts immune responses. The production of EPS is tightly regulated and often coordinated by QS systems, making these two processes intricately linked.

Fucoidan is a sulfated polysaccharide, numerous algal species generated in large quantities, and its biological characteristics have been thoroughly studied [12,13,14]. By altering the permeability of the cell wall or creating a gel-like barrier on the bacterial membrane, fucoidan is thought to have antibacterial properties that prevent bacterial nutrition exchange and suppress bacterial pathogens [15,16,17]. The search for new antimicrobial agents has been spurred by the growing resistance of microorganisms to traditional antibiotics [18,19]. Because of their alleged less adverse effects and affordability, natural substances like fucoidan hold promise as possible sources of antimicrobial medications [9,11]. Emerging evidence indicates that fucoidan may also disrupt biofilm formation and reduce the stability of pre-formed biofilms in various microbial species, making it a potential agent for combating biofilm-related infections [20,21]. However, its efficacy in the reduction in *Salmonella enterica* subsp. *enterica* Serovar *Typhimurium* biofilms on seafood surfaces, such as crab and shrimp, remains largely unexplored.

This study aims to assess the impacts of fucoidan of sub-minimum inhibitory concentrations (sub-MIC) in preventing *Salmonella enterica* subsp. *enterica* Serovar *Typhimurium* biofilm formation on crab and shrimp and its influence on the expression of key genes at the molecular level. The results will offer effective perceptions of the possible use of fucoidan as an antimicrobial agent in the seafood industry, enhancing food safety and minimizing microbial contamination.

## 2. Materials and Methods

### 2.1. Bacterial Strain Culture and Growth

In this study, *Salmonella enterica* subsp. *enterica* Serovar *Typhimurium* (*S*. *Typhimurium* ATCC 13311) was utilized as the test organism. The bacterial strains, with a cell density of 10^7^–10^8^ Log CFU/mL, were stored at −80 °C in tryptic soy broth (TSB; BD Difco, Franklin Lakes, NJ, USA) accompanied by 30% glycerol. Initially, 100 μL of the bacterial stock was inoculated and incubated at 37 °C into 10 mL of TSB with continuous shaking at 170 rpm using a shaking incubator (Vision Scientific, VS–8480, Seoul, Republic of Korea). A 100 μL aliquot was subsequently transferred into 10 mL of TSB and incubated for 24 h. After 18 h incubation, the bacterial culture was centrifuged at 5400 rpm for 10 min at 4 °C, washed two times with sterile phosphate-buffered saline (PBS; Sigma-Aldrich, St. Louis, MO, USA), and resuspended in the same buffer. The final suspension was then diluted using peptone water (PW) (Oxoid, Basingstoke, UK) to accomplish 10^5^ Log CFU/mL. This prepared inoculum (10^5^ Log CFU/mL) was used to facilitate biofilm formation on crab and shrimp.

### 2.2. Fucoidan and Minimum Inhibitory Concentration (MIC) Determination

In this study, fucoidan was obtained from Sigma-Aldrich (St. Louis, MO, USA). The compound was dissolved in DW at 1 mg/mL concentration. The MIC of fucoidan against *S*. *Typhimurium* ATCC 13311 was determined by a previous method [22]. A two-fold serial dilution technique was employed to establish the MIC in TSB. In a 96-well plate (Corning Incorporated, Corning, NY, USA), 100 µL of fucoidan (0–300 µg/mL) was diluted serially in TSB and mixed with 100 µL of a bacterial suspension (10^5^ Log CFU/mL), resulting in 200 µL per well. The cultures were incubated at 37 °C for 24 h and bacterial growth was assessed by evaluating absorbance at 600 nm using a microplate reader (Spectra Max 190, Sunnyvale, CA, USA). After overnight incubation, 100 µL aliquots from wells showing no visible growth were plated on xylose lysine deoxycholate (XLD) agar (Thermo Scientific, Oxoid, Basingstoke, UK) to enumerate bacterial colonies. The experiment was performed three times, and the MIC was revealed to be 150 µg/mL of fucoidan. For subsequent experiments, the sub-MIC concentrations (0, 1/8, 1/4, 1/2, and MIC) of fucoidan were utilized.

### 2.3. Crabs and Shrimp Sample Preparation

With minor changes, sample preparation was conducted following methods described in previous studies [23]. Shrimp (*Penaeus monodon*) and crab (*Corystes cassivelaunus*) were purchased from the local market and with a sterile scalpel, they were cut into 2 × 2 cm^2^ coupons. Any residual meat was removed, and the shells were thoroughly washed with sterile DW. To eliminate background microflora on each side of the coupons, the coupons were exposed to UV-C light for 15 min before bacterial inoculation. The prepared coupons were then submerged into 10 mL of TSB in a 50 mL Falcon tube, inoculated with *S*. *Typhimurium* ATCC 13311 (10^5^ Log CFU/mL), and incubated at 37 °C for 24 h under static conditions for experimentation.

### 2.4. Biofilm Formation Process

The technique was conducted with minor modifications from a previous method [24]. Biofilm inhibition was observed at sub-MIC and MIC, which may not have directly eliminated the bacteria but potentially influenced their virulence factors. The experimental setup included a control (without fucoidan) and fucoidan concentrations of 1/8, 1/4, 1/2, and MIC.

Fucoidan at the designated concentrations (0, 1/8, 1/4, 1/2, and MIC) was added to 10 mL of TSB in 50 mL Falcon tube with food surface samples. A 100 µL suspension of bacteria (10^5^ Log CFU/mL) was introduced, and the mixture was homogenized by a vortex mixer (Scientific Industries, SI-0256, Bohemia, NY, USA). The samples were incubated at 37 °C for 24 h to allow biofilm development.

Following biofilm formation, loosely attached bacteria were removed by washing the coupons twice with DW. The surfaces were then immersed in 10 mL of PW (containing 10 sterile glass beads) and vortexed for 2 min to detach adherent bacteria. The recovered bacterial suspension was serially diluted and plated on XLD to assess bacterial colony counts. After incubation at 37 °C for 24 h, CFU were enumerated. Biofilm inhibition was quantified by calculating the colony of bacteria in bacterial populations (Log CFU/cm^2^) at each fucoidan concentration compared to the control.

### 2.5. Biofilm Visualization by Field Emission Scanning Electron Microscopy (FE-SEM)

To visually confirm biofilm inhibition by fucoidan (at concentrations of 0, 1/8, and 1/2 MIC) on crab and shrimp coupons, FE-SEM was employed. The sample preparation procedure was adapted with minor adjustments from the previous protocol [25]. In brief, the crab and shrimp coupons were fixed in 2.5% glutaraldehyde in PBS and stored at room temperature for 4 h. Following fixation, the coupons were subjected to ethanol treatments (50%, 60%, 70%, 80%, and 90%) for 15 min each and two times in 100% ethanol immersions.

The samples were then dehydrated by sequential soaking in increasing concentrations of hexamethyldisilazane (33%, 50%, 66%, and 100% in ethanol) for 15 min each. After dehydration, the samples were air-dried in a fume hood for 3 h and coated with platinum using a sputter coater (Q150T Plus, Quorum, UK) and examined using a FE-SEM (Hitachi/Baltec S-4700, Tokyo, Japan) to observe the biofilm morphology.

### 2.6. Relative Expression of Gene by Real-Time PCR (RT–PCR)

This test was conducted with minor modifications based on a previously described protocol [26,27] to analyze the impact of fucoidan on the expression of quorum-sensing and virulent genes in *S*. *Typhimurium* ATCC 13311. Fucoidan-treated bacterial suspensions (10^5^ log CFU/mL) were inoculated into a Falcon tube (10 mL of TSB) and incubated at 37 °C for 24 h to facilitate biofilm formation. After the incubation, RNA was extracted from the bacterial pellet using the RNeasy Mini kit (Qiagen, Hilden, Germany), following centrifugation.

The RNA concentrations and purity were assessed using a spectrophotometer (NanoDrop, Bio-Tek Instruments, Chicago, IL, USA) at absorbance ratios of 260/280 nm and 260/230 nm. Complementary DNA (cDNA) was synthesized using the Maxime RT PreMix kit (iNtRON Biotechnology Co., Ltd., Seoul, Gyeonggi-do, Republic of Korea). Primers used for the RT-qPCR analysis are listed in Table 1, with 16S rRNA serving as the housekeeping gene.

For quantitative PCR (RT-qPCR), a 20 µL reaction mix containing 1 μL of cDNA, appropriate primers, and Power SYBR Green PCR Master Mix (Applied Biosystems, Thermo Fisher Scientific, Warrington, UK) was prepared. RT-qPCR was performed with the following thermal cycling conditions: initial denaturation at 95 °C for 20 s, followed by annealing at 50 °C for 20 s, and extension at 72 °C for 20 s. Melting curve analysis was performed to confirm specificity, and gene expression was analyzed using the 2^−△△Ct^ method.

### 2.7. Statistical Analysis

Each experiment was performed in triplicate. The results are presented as the mean ± standard error of the mean (SEM). One-way analysis of variance (ANOVA), followed by Duncan’s multiple range test, was analyzed to assess significance. Statistical significance was considered as *p*-value (<0.05). The data were evaluated using SAS software version 9.2 (SAS Institute Inc., Cary, NC, USA).

## 3. Results

### 3.1. MIC Determination

**Figure 1:**
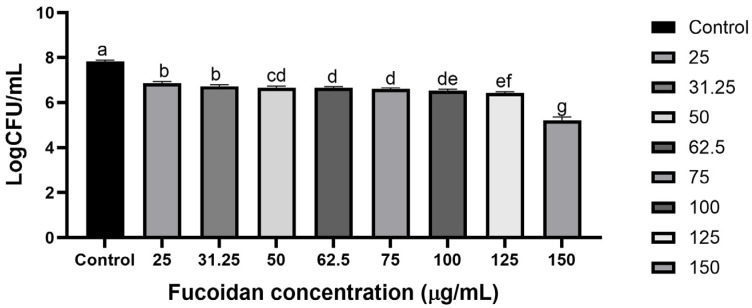
Minimum inhibitory concentrations of Fucoidan against *S*. *Typhimurium* ATCC 13311. Data are presented as mean ± SEM from three independent experiments. Within each treatment, values with different letters (a–g) indicate significant differences according to Duncan’s multiple range test (*p* < 0.05).

Fucoidan’s inhibitory effect was found to be dose-dependent and varies across different species. MIC was defined as the lowest concentration of fucoidan at which no visible bacterial growth was observed. To evaluate its antimicrobial activity against *S*. *Typhimurium* ATCC 13311, further experiments were conducted to determine the MIC of fucoidan. With fucoidan concentrations (ranging from 0 to 150 µg/mL), a significant effect on bacterial growth was observed up to 150 µg/mL (*p* ≥ 0.05) (Figure 1). Based on these results, the sub-MIC range was established as 31.25–125 µg/mL, which was used in all subsequent experiments. The MIC of fucoidan against *S*. *Typhimurium* ATCC 13311 was revealed at 150 µg/mL. Sub-MIC and MIC concentrations (1/8, 1/4, 1/2, and MIC) were employed for further investigations.

### 3.2. Eradication Impact of Fucoidan on Crab and Shrimp Against S. Typhimurium ATCC 13311

**Figure 2:**
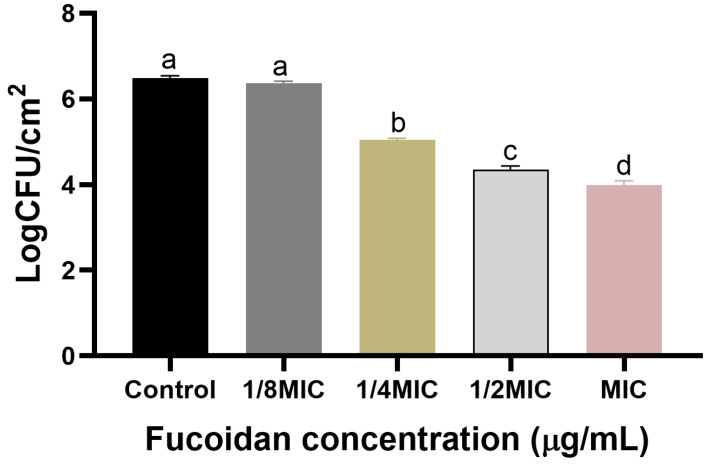
Biofilm formation by *S*. *Typhimurium* ATCC 13311 on crab coupons in the presence of sub-MIC of fucoidan (0, 1/8, 1/4, 1/2, and MIC) under optimal conditions (37 °C, pH 7.0). Data are expressed as mean ± SEM from three independent replicates. Within each treatment, values with different letters (a–d) indicate significant differences according to Duncan’s multiple range test (*p* < 0.05).

**Figure 3:**
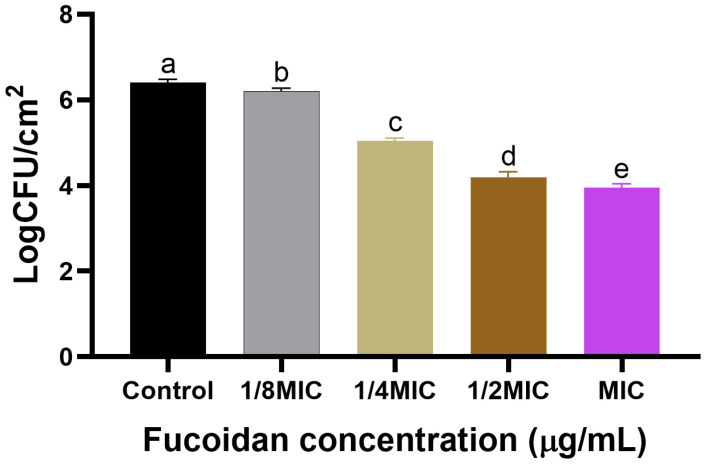
Biofilm formation by *S*. *Typhimurium* ATCC 13311 on shrimp coupons in the presence of sub-MIC of fucoidan (0, 1/8, 1/4, 1/2, and MIC) under optimal conditions (37 °C, pH 7.0). Data are expressed as mean ± SEM from three independent replicates. Within each treatment, values with different letters (a–e) indicate significant differences according to Duncan’s multiple range test (*p* < 0.05).

The biofilm formation of *S*. *Typhimurium* ATCC 13311 on crab and shrimp coupons was effectively inhibited by fucoidan, as demonstrated in Figure 2 and Figure 3, respectively. As the concentration increased, the inhibition of biofilm also showed a corresponding increase. On crab surfaces, the biofilm inhibition values of *S*. *Typhimurium* ATCC 13311 were 0.12, 1.45, 2.13, and 2.50 log CFU/cm^2^ at fucoidan concentrations of 1/8, 1/4, 1/2, and MIC, respectively (Figure 2). Similarly, on shrimp coupons, the biofilm inhibition values were 0.19, 1.36, 2.21, and 2.45 log CFU/cm^2^ at fucoidan concentrations of 1/8, 1/4, 1/2, and MIC (Figure 3).

### 3.3. Confirmation of Biofilm Reduction Visually by Fucoidan

**Figure 4:**
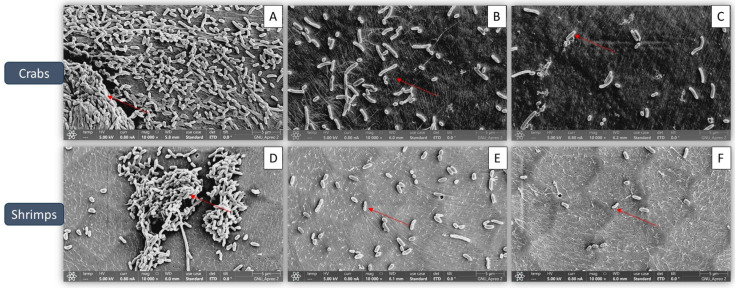
Representative scanning electron micrographs of *S*. *Typhimurium* ATCC 13311 biofilm formation on crabs and shrimp under optimal conditions in the presence of different sub-MIC concentrations of fucoidan: (**A**) control; (**B**) 1/8 MIC; (**C**) 1/2 MIC for crabs, and (**D**) control; (**E**) 1/8 MIC; (**F**) 1/2 MIC for shrimp.

Figure 4 presents the visual validation of biofilm inhibition by fucoidan. In the control samples, the biofilms exhibited well-organized architectural structures with intact cell-to-cell contacts, with smooth and regular cells having intact cell membranes (Figure 4A,D). In contrast, the fucoidan-treated samples displayed bacterial cells with a rough and irregular appearance, indicating a loss of their normal shape (Figure 4C,F). The red color marks in the control samples (Figure 4A,D) indicate the attachment of biofilm cells, while in the fucoidan-treated groups, the biofilms showed significant lysis, as evidenced by the reduced presence of red-stained cells (Figure 4B,C,E,F).

### 3.4. Relative Expression Levels

**Figure 5:**
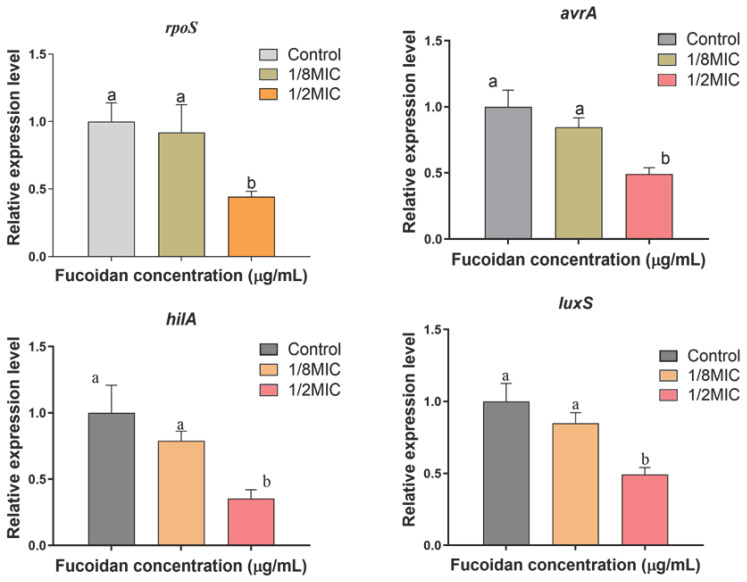
Relative expression levels of *rpoS*, *avrA*, *hilA*, and *luxS* genes in *S*. *Typhimurium* ATCC 13311 suspension supplemented with different concentrations of fucoidan. Different superscript letters (a, b) indicate significant differences (*p* < 0.05) based on three independent replicates.

Figure 5 shows the levels of relative expression of virulence (*rpoS*, *avrA*, and *hilA*) as well as the QS gene (*luxS*) in *S*. *Typhimurium* ATCC 13311, measured by qPCR in the presence of sub-MIC of fucoidan (ranging from 0 to 125 µg/mL). Gene expression was significantly downregulated at all sub-MIC concentrations of fucoidan (*p* < 0.05).

## 4. Discussion

This study explored the efficacy of fucoidan in biofilm inhibition of *S*. *Typhimurium* ATCC 13311 on crab and shrimp and provided insights into its potential mechanisms of action. The findings demonstrated that fucoidan, even at sub-MICs, significantly reduced biofilm formation and destabilized pre-formed biofilms. Fucoidan, a sulfated polysaccharide derived from *Fucus vesiculosus*, exhibited antimicrobial properties against food-pathogenic bacteria [28,29]. In previous studies, the MICs ranged from 125 to 1000 µg/mL [30]. Fucoidan concentrations exceeding 250 µg/mL were highly effective in inhibiting biofilm formation and planktonic cell growth of *Streptococcus mutans* and *S. sobrinus* [30]. These results underscore the potential application of fucoidan as a natural antimicrobial and antibiofilm agent in the seafood industry.

Biofilm growth contributes to ongoing contamination and resistance to traditional cleaning techniques, it presents serious problems for the seafood sector [31]. According to the current study, fucoidan decreased *S*. *Typhimurium* ATCC 13311 ability to form biofilms on crabs and shrimp in a concentrate-dependent manner, with significant reductions shown at 1/2 MIC and MIC concentrations (Figure 1). Previous investigations have documented similar antibiofilm activities of fucoidan against various pathogens, such as *Staphylococcus aureus* and *Escherichia coli* [32]. The 150 µg/mL MIC value for *S*. *Typhimurium* ATCC 13311 found in this investigation is consistent with reports of fucoidan’s antibacterial effectiveness against Gram-negative bacteria. For instance, MIC values for *Pseudomonas aeruginosa* and *E. coli* ranged from 100 to 200 µg/mL [33]. Together, these results demonstrate the broad-spectrum potential and dose-dependent nature of fucoidan’s antibacterial qualities. Its promise as a food-safe antimicrobial intervention is highlighted by the observed decreases in biofilm-associated bacterial counts, which were as high as 2.50 log CFU/cm^2^ on crab coupons and 2.45 log CFU/cm^2^ on shrimp coupons. In a previous study, fucoidan F85 was tested at concentrations ranging from 0 to 250 µg/mL. The growth control group in BHI broth increased from approximately 5.2 log CFU/mL (initial cell count) to around 8.6 log CFU/mL after 18 h, maintaining this level with a slight decline of about 1 log cycle after 48 h. In contrast, *S. mutans* treated with fucoidan at 125 µg/mL showed no growth, while *S*. *mutans* treated with fucoidan F85 at 250 µg/mL exhibited a consistent decrease, reaching approximately 3.3 log CFU/mL after 48 h [30]. FE-SEM imaging provides evidence of fucoidan’s mode of action by identifying notable structural anomalies in individual bacterial cells and the biofilm matrix. The smooth and undamaged looks of untreated control cells were sharply contrasted with the rough and uneven surfaces of bacterial cells in fucoidan-treated samples. These results align with earlier research showing that fucoidan breaks down the EPS matrix, which is essential for preserving the structure of biofilms and promoting bacterial adhesion [34]. In addition to this experiment, virulence-associated genes (*rpoS, hilA, avrA*, and *luxS*) were shown to be significantly downregulated in *S*. *Typhimurium* ATCC 13311 biofilms treated with fucoidan. These genes are important for bacterial communication, biofilm control, and pathogenicity; fucoidan may interfere with these functions to prevent the formation of biofilms [35]. In the present study, the application of sub-inhibitory concentrations of fucoidan significantly reduced biofilm formation on various seafood coupons. This antibiofilm activity may be attributed to the disruption of QS signaling and EPS production. Gene expression analysis revealed significant downregulation of *luxS* in *S*. *Typhimurium* ATCC 13311, indicating a possible QS-inhibitory effect of fucoidan. Additionally, genes associated with virulence and stress response (*hilA*, *avrA*, *rpoS*) were also suppressed, further suggesting that fucoidan interferes with key regulatory pathways involved in biofilm development and maintenance.

By inhibiting QS and reducing EPS synthesis, fucoidan compromises biofilm integrity and diminishes the protective advantages conferred by the biofilm matrix. These findings highlight fucoidan’s potential as a natural QS and EPS-targeting agent, offering an effective strategy to combat persistent biofilms in food industry settings.

All of the observed decreases in gene expression point to fucoidan’s complex antibiofilm mechanisms. Inhibiting biofilm formation and destabilizing pre-existing biofilms, fucoidan targets quorum-sensing, stress response, and virulence pathways. These results are similar to previous studies that suggested natural polysaccharides might function as potent antibiofilm agents by interfering with molecular pathways [36,37].

The seafood industry faces significant challenges with biofilm-associated contamination, which poses serious food safety and public health concerns. According to this study, fucoidan shows promise as a natural remedy for these problems. Fucoidan, which comes from brown algae, has several benefits over conventional chemical disinfectants, such as being naturally derived, having minimal toxicity, and being environmentally friendly [28,38]. Additionally, its effectiveness at sub-MIC levels increases its applicability by cutting costs in industrial applications and lessening the danger of the development of antibiotic resistance [39]. Sensory evaluation was conducted on butter samples from both control and treatment groups, including varying fucoidan concentrations over different storage durations [40]. The results demonstrated that butter samples containing 0.5% fucoidan exhibited significantly reduced acid and peroxide values, extending shelf life as supported by sensory assessments [41]. No alterations in taste were detected in any samples on day zero, and even at higher fucoidan concentrations, no adverse effects on flavor were observed. An increase in fucoidan concentration resulted in slightly lighter butter color and enhanced texture characteristics, such as improved softness and spreadability. By day 40, a slight increase in rancid flavor was noted across all samples; however, the control group displayed the most pronounced rancidity, whereas the samples treated with the highest fucoidan concentrations maintained acceptable sensory quality without unpleasant organoleptic changes [42]. Shelf life, defined as the duration during which a food product remains acceptable in terms of safety, nutrition, and sensory attributes, was notably prolonged in the fucoidan-treated samples.

However, this study also points to several limitations. The effectiveness of fucoidan has to be confirmed in actual seafood processing settings, as the trials were carried out in a controlled laboratory setting. To guarantee its usefulness in the sector, research should also be carried out on its affordability and any effects on the sensory aspects of fish products. While this study highlights the promising antibiofilm and antimicrobial properties of fucoidan against *S*. *Typhimurium* ATCC 13311 on seafood coupons, translating these findings into industrial application necessitates careful consideration of several practical factors. Regulatory approval is a foremost concern; although fucoidan is derived from edible brown seaweed and is generally regarded as safe (GRAS) in some jurisdictions, its application as an antimicrobial agent in food processing environments would require compliance with food safety regulations governed by authorities such as the FDA, EFSA, or regional food safety agencies. Comprehensive toxicological assessments and efficacy validations under industrial conditions would be essential for its approval.

Moreover, sensory impacts on seafood products must be thoroughly evaluated. Even though fucoidan is considered low in toxicity and natural in origin, its potential influence on the organoleptic properties—including taste, texture, aroma, and appearance—of treated seafood could affect consumer acceptability. It is imperative that sensory testing be integrated into future studies to ensure the maintenance of product quality post-treatment.

Formulation and application challenges also merit close attention. Fucoidan’s stability under various processing conditions—such as temperature, pH, and exposure to other food-grade chemicals—must be addressed. Effective delivery mechanisms, such as incorporation into edible coatings, sprays, or wash solutions, should be optimized for uniform application and maximal biofilm inhibition. Furthermore, large-scale production of standardized fucoidan with consistent bioactivity is necessary to ensure batch-to-batch reproducibility, a requirement for commercial scalability.

In addition, although fucoidan holds significant promise as a natural, eco-friendly antimicrobial in the seafood industry, its practical implementation demands multi-faceted exploration involving regulatory, sensory, and formulation studies. Addressing these challenges through interdisciplinary research will be critical to unlocking fucoidan’s full potential in enhancing food safety and reducing microbial contamination.

Fucoidan’s synergistic effects with other natural antimicrobials should be investigated in future studies to increase its effectiveness against infections linked with biofilms.

The study’s results imply that fucoidan may be used as a safe and efficient natural antibiofilm agent to combat *S*. *Typhimurium* ATCC 13311 on seafood coupons. It offers a viable method for enhancing food safety and minimizing microbial contamination in the seafood sector because of its capacity to prevent the creation of new biofilms, decrease existing biofilms, and alter quorum-sensing pathways. To maximize its usage and broaden its application to additional foodborne diseases and industrial settings, more research is necessary.

## 5. Conclusions

Fucoidan demonstrates strong antimicrobial and antibiofilm activity against *S*. *Typhimurium* ATCC 13311 on seafood coupons, significantly reducing bacterial counts by 2.50 log CFU/cm^2^ on crabs and 2.45 log CFU/cm^2^ on shrimp at sub-MIC levels. FE-SEM analysis confirmed its ability to disrupt the EPS matrix, likely by interfering with quorum sensing and virulence gene expression. With its natural origin, low toxicity, and effectiveness at sub-MIC levels (31.25–125 µg/mL), fucoidan presents a sustainable alternative to chemical disinfectants in the seafood industry. Further research is needed to assess its industrial application, potential synergistic effects, and impact on product quality, making it a promising tool for enhancing food safety and reducing microbial contamination.

## Figures and Tables

**Table 1:** Names of primers related to virulence and QS.

Target Primers	Sequence (5′–3′)	Product Size (bp)
*16S rRNA*	F: CAGAAGAAGCACCGGCTAAC	167
	R: GACTCAAGCCTGCCAGTTTC	
*rpoS*	F: GAATCTGACGAACACGCTCA	171
	R: CCACGCAAGATGACGATATG	
*avrA*	F: GAGCTGCTTTGGTCCTCAAC	173
	R: AATGGAAGGCGTTGAATCTG	
*hilA*	F: ATTAAGGCGACAGAGCTGGA	134
	R: GCAGAAATGGGCGAAAGTAA	
*LuxS*	F: CGGGTTGCAAAAACGATGA	150
	R: GTTGAGGTGGTCGCGCATA	

## Data Availability

The original contributions presented in this study are included in the article. Further inquiries can be directed to the corresponding authors.
